# Applications of Delayed Fluorescence from Photosystem II

**DOI:** 10.3390/s131217332

**Published:** 2013-12-16

**Authors:** Ya Guo, Jinglu Tan

**Affiliations:** Department of Bioengineering, University of Missouri, Columbia, MO 65211, USA; E-Mail: tanj@missouri.edu

**Keywords:** delayed fluorescence, delayed light, delayed luminescence, chlorophyll fluorescence, plant stress, environmental pollution, photosynthesis

## Abstract

While photosystem II (PSII) of plants utilizes light for photosynthesis, part of the absorbed energy may be reverted back and dissipated as long-term fluorescence (delayed fluorescence or DF). Because the generation of DF is coupled with the processes of forward photosynthetic activities, DF contains the information about plant physiological states and plant-environment interactions. This makes DF a potentially powerful biosensing mechanism to measure plant photosynthetic activities and environmental conditions. While DF has attracted the interest of many researchers, some aspects of it are still unknown because of the complexity of photosynthetic system. In order to provide a holistic picture about the usefulness of DF, it is meaningful to summarize the research on DF applications. In this short review, available literature on applications of DF from PSII is summarized.

## 1. Introduction

### 1.1. Background

**Figure 1.**
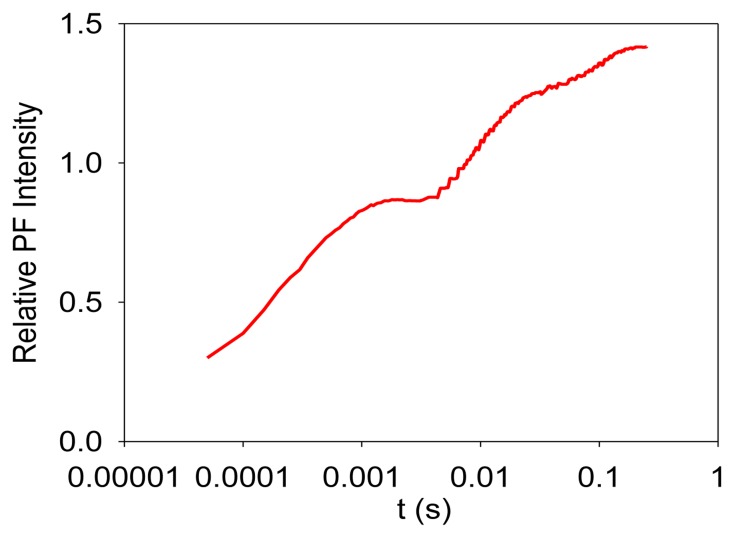
A measured PF signal.

**Figure 2.**
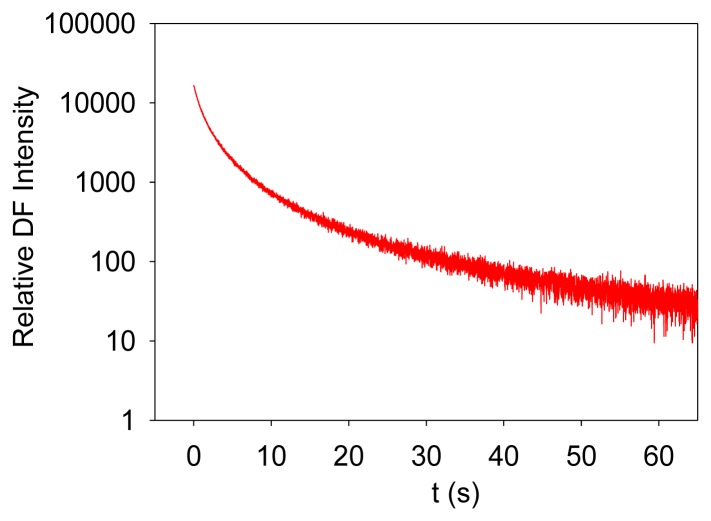
A measured DF signal.

Photosynthesis is the only known biological process that can harvest sun light energy [1]. Life on Earth ultimately relies on the harvested energy to drive all kinds of activities. Oil, gas, and coal are all products from photosynthesis, which are essential to modern industry [2]. The oxygen produced from photosynthesis allows aerobic life to exist [3,4]. Photosynthesis-related research is thus very important. Photosynthesis starts from light-induced separation of charge pairs and transport of electrons by various electron carriers [1,5]. When a photo-excited electron of a chlorophyll molecule in photosystem II (PSII) returns to the ground state, a new photon is regenerated, which is commonly referred to as fluorescence or prompt fluorescence (PF). The PF usually has a lifetime in the order of pico- or nanoseconds. The excited electron can also be transferred forward and used for photochemical reactions. Because chemical reactions are usually reversible, this electron can be transferred back, resulting in chlorophyll molecules (e.g., P680 or PSII antenna chlorophyll molecules) in the excited state, and capable of emitting chlorophyll fluorescence. After the excitation light is turned off, there are excited electrons along the electron transport chain. It takes time for these electrons to transfer back to generate chlorophyll fluorescence. This type of fluorescence thus has a much longer lifetime (minutes or even hours) and is commonly called delayed fluorescence (DF, also known as delayed light, delayed luminescence or DL) [6–10]. Examples of measured PF and DF from PSII are shown in Figures 1 and 2, respectively.

It is worth mentioning that PF is measured when excitation light is turned on, and it shows a trend of increasing with recording time before light adaptation. DF is measured when the excitation light is turned off, and it thus shows a trend of decreasing with recording time. DF is not measurable when the excitation light is turned on because PF and DF have the same wavelength, but PF is much stronger than DF, and the excitation light may damage the high-sensitive DF measurement setup.

Because the emissions of DF and PF are associated with forward photosynthetic reactions, PF and DF from PSII can be used as indicators of plant physiological states and environmental changes. Applications of PF have been well summarized in the literature [11–15], but there is a lack of a review of DF applications. This short review summarizes available literature that focused on applications of DF from PSII. It is hoped that this will serve as a useful reference for future research on developing DF-based sensing techniques for plant production, environmental monitoring and other application. It should note that the focus of this work is on DF applications. For other aspects of DF, the reader is referred to existing reviews. For example, an overview of DF can be found in [16], a report on the DF from PSII and Photosystem I is given in [17], and the relationship between PF and DF is discussed by Lavorel [18], Amesz and Van Gorkom [19], Malkin [20], Lavorel *et al.* [21], Jursinic [22], Radenovic *et al.* [23], and Tyystjarvi and Vass [24].

### 1.2. Factors Affecting DF Emission

DF emission displays multiple phases over the emission time span [25]. These phases correspond to different components of the electron transport chain. The exact mechanism for the final DF emission is not yet fully understood. There are different explanations [16,19], including the electron-hole recombination theory [26–28], the triplet fusion theory [29,30], and the charge-recombination theory [22,31–34]. The charge-recombination theory is more widely accepted [26–28,33,35]. The exact mechanism, however, does not alter the fact that the exited electrons come from different stages of the electron transport chain. A variable that influences PSII functions may affect DF emission and thus can be potentially indicated by DF. PSII functions are affected by many factors. Some interesting factors are listed below:

(1) *Water*. Water provides electrons to return oxidized P680^+^ to its reduced form P680 [1,2] and supports many other functions of plant life. DF emission is thus affected by the water status of plants [36].
(2) *Chlorophyll concentration*. Chlorophyll molecules serve as PSII antenna complexes [1,2]; therefore, their concentration determines the absorbed light and influence DF emission.
(3) *Herbicides*. Most herbicides function by binding to certain sites of PSII and impeding electron transport, and thus influence DF emission [1,7,37].
(4) *Heavy metals*. Heavy metals like copper, mercury, and lead may accumulate in plants over time. Some research indicates that PSII is sensitive to heavy metal contents [38].
(5) *pH and temperature*. Both affect chemical reaction speed and chemical balance [2,39], and thus influence DF emission.
(6) *Excitation-light wavelength and intensity*. Light drives photosynthesis and thus has a significant impact on DF emission [40,41].
(7) *Nutrient status*. Nutrient status affects plant photosynthesis rate and chlorophyll concentration and thus affects DF emission [42].

Because DF can be potentially very useful, it has attracted extensive attention from researchers. In this paper, DF applications reported in the literature are concisely summarized, including measurement or assessment of photosynthesis rate, plant circadian, plant senescence, nutrients, salt stress, chilling stress, heat stress, acid rain, herbicide, metal pollution, aquatic ecosystems, and drought stress.

## 2. DF Applications

### 2.1. Photosynthesis Rate

Measurement of photosynthesis is very important for evaluating plant physiology and crop monitoring. Photosynthesis research used to be dominated by gas-exchange measurements, which include sophisticated (and costly) systems for simultaneously detecting CO_2_ uptake and H_2_O evaporation [43,44]. The advent of DF-based techniques provides alternative ways for measuring photosynthesis rate. Xing and coworkers developed DF-based system for photosynthesis rate measurement based on the correlation between DF and photosynthesis efficiency [40,45,46]. Wang *et al.* described a DF-based photosynthesis rate measurement setup with DF intensity as a measure. The setup could be used in different weather conditions [40]. Wang *et al.* further demonstrated the reliability of the DF-based photosynthesis rate measurement setup. When the DF-based results were compared with the results measured through a well-known commercial photosynthesis measurement system (LI-6400, LI-COR, Lincoln, NE, USA), the uncertainty was less ±5% [45]. In [46], the DF-based photosynthesis rate measurement setup was improved and could be used for non-invasive and real-time measurement of photosynthetic metabolism regulations. Experiments indicated the setup could accurately measure the effects of NaHSO_3_ on photosynthetic metabolism. Xu and Li tried to reduce power consumption of DF-based photosynthetic capacity measurement through unsaturated light excitation. Experimental results in this work demonstrated that there was an excellent linear relationship between photosynthesis rates and the intensity of DF signal [47]. Björn and Forsberg developed an apparatus for obtaining DF images of plants. The apparatus consists of a phosphoroscope, imaging lens, an electronic image intensifier, and light sources. Images acquired with the apparatus could show damages of photosynthetic system caused by virus and insects [48]. The existing work indicates that DF is a useful indictor of photosynthesis rate.

### 2.2. Plant Circadian

The plant circadian clock plays a vital role in enhancing photosynthesis performance and regulating plant growth. Studies of the clock are limited by the lack of a simple, accurate, and robust circadian indicator [49]. Kurzbaum *et al.* compared DF excitation spectroscopy with radiocarbon technique using a monoalgal culture of *Chlorella vulgaris* grown under natural temperature and irradiance. In the experiments, the samples were kept in natural irradiance right after the DF measurement. They found that DF signal correlated with both the quantum efficiency and radiant energy utilization efficiency during a diurnal cycle. The intensity of DF oscillates with an approximately the 24-h period of the circadian clock and can thus be used as an indicator of plant circadian [50]. DF-based plant circadian measurement brings a lot of hope for future plant circadian and plant metabolism research.

### 2.3. Plant Senescence

During senescence, plant leaves experience many metabolic changes, which result in declining photosynthetic activities and chloroplast functions. Because senescence limits crop yield, detecting plant senescence is very meaningful to agriculture production. Most commercial senescence measurement instruments are still gas-exchange-based and the accuracy is subject to environmental variations [51]. Because DF is a good indicator of photosynthetic activities, it should be useful for sensing plant leaf senescence. Zhang *et al.* measured DF, chlorophyll content, ion leakage, and net photosynthesis rate based on the consumption of CO_2_ for leaves with various senescence symptoms induced by age or hormones. It was found that the changes in DF could reflect the changes in photosynthetic capacity and chlorophyll content during age-dependent and hormone-modulated senescence [52]. This work indicated that DF could be used to sense plant senescence. Experiments demonstrated that DF emission correlated well with photosynthesis rate and typical plant senescence symptoms such as chlorophyll reduction and ion leakage. Compared with traditional methods based on gas exchange techniques and biochemical assays, DF-based techniques are easy to operate and can be used under different weather conditions.

### 2.4. Nutrients

Balanced nutrients are essential to plant growth [53]. Measurement of plant nutrient status is becoming increasingly vital as the world turns to biomass as a major source of energy; but there has been a lack of effective methods to detect plant nutrient limitations. For example, reliable and rapid techniques are wanted for investigating phytoplankton physiology [54]. Because of the close relationship between DF and photosynthesis, DF may be a potential sensor for plant nutrient status measurement. In [55], Bürger and Schmidt found that DF of green plants ranging from 0.3 s up to several minutes was highly dependent on nutritional deficiencies, and that DF of the unicellular green alga, *Scenedesmus obliquus*, was strongly affected by depletion of the growth medium of various essential elements such as N, Fe, Ca, Mg or K. This indicated that DF could be a convenient, highly sensitive, and specific assay for a number of nutrients. The work of Berden-Zrimec *et al.* confirmed this conclusion, in which, the marine unicellular alga *Dunaliella tertiolecta Butcher* (*Chlorophyta*) was used and DF intensity was compared with cell concentration and chlorophyll a fluorescence [42].

### 2.5. Salt Stress

Salt stress reduces the rates of photosynthesis. It is a major environmental factor that limits plant productivity. A biosensor that is capable of measuring salt stress is very important. In [56,57], Zhang and Xing studied the photosynthetic activities of spinach leaves directly exposed to different NaCl concentrations. Their experiments suggested that DF had an excellent correlation with photosynthesis rate under salt stress and could be used as a sensitive test for plant salt stress. Mehta *et al.* studied the changes in the heterogeneity of PSII by measuring PF and DF from wheat leaves after salt treatment. The measured DF provided information about the change in the antenna size heterogeneity of PSII [58]. The work further indicated that DF could be used for measurement of salt stress.

### 2.6. Chilling Stress

Chilling stress is very common to plants. Evaluation of chilling stress is meaningful for crop yield prediction, chilling-resistant species selection, and chilling-resistant species culturing. DF was found highly dependent on temperature and chilling stress [59–62]. There was a linear relationship between DF intensity and temperature, but the DF intensity of chilling-sensitive species and that of chilling-insensitive species were affected by temperature differently [63]. DF intensity from chilling-sensitive maize at steady-state level showed a maximum near the temperature at which thylakoid membrane lipids undergo a phase transition; on the other hand, DF emission from chilling-resistant barley did not show phase transition above 0 °C and the DF intensity only varied in a monotonic fashion [64]. In [65], Abbott *et al.* studied the effects and chilling stress on DF emission and found that the major DF peak was greatly inhibited in the chilling susceptible species, but showed only small changes in the chilling tolerant species. These findings show that DF *in vivo* can offer a rapid and sensitive method for chilling stress detection and chilling-resistant species selection.

### 2.7. Heat Stress

Heat stress has been a major environmental factor that affects plant growth and productivity. High temperature affects the rate of chemical reactions and structural organization [66]. The effect of heat stress on DF emission was well observed in previous research. By using a lamina of soybean as a testing model, Zeng and Xing investigated the effects of heat stress on plant photosynthesis capability. Experimental results showed that DF spectrum could be potentially useful for characterizing the changes of soybean photosynthesis capability under different degrees of heat stress and it might be a rapid approach for detecting heat stresses [67]. Spectroscopy measurements reported by Li *et al.* indicate that heat stress influences the shape of DF emission spectra of C3 soybean (*Jing Huang No. 3*) and C4 maize (*Yun Xi No. 5081*) [66]. In other work, Oukarroum *et al.* observed DF from pea leaves changing with temperature under heat stress (25–50 °C) [68]. DF was also used as a method to select heat-stress-resistant species. In [69], Zhang *et al.* developed a biosensor for identifying high-temperature-resistant species based on DF measurement. The portable biosensor used light-emitting diode lattice as excitation light source and could detect DF emission *in vivo.* Measurements with the developed sensor demonstrated that DF intensity correlated with photosynthesis rate under heat stress and it provided a reliable approach for rapid and non-invasive determination of heat stress effects.

### 2.8. Acid Rain

Acid rain is believed to result from emissions of sulfur dioxide and nitrogen oxide from human activities. It has an impact on the structure and function of chloroplast in plant leaves and thus affects photosynthesis and crop production. Finding a plant-physiology-based measurement for the effects of acid rain is very desirable for agriculture. By using zijinghua (*Bauhinia variegata L*.) and soybean (*Glycine max (L.) Merr*.), Wang *et al.* studied the effects of artificial acid rain and SO_2_ on DF emission. They found that changes in DF intensity can reflect chloroplast intactness and function as affected by acid stress [51]. In [70], Zeng and Xing developed a DF-based biosensor that can inspect acid rain pollution *in vivo.* Compared with traditional methods, the developed DF-based biosensor can continuously monitor acid stress and is not affected by weather conditions.

### 2.9. Herbicide

While the application of herbicides increases the yields of modern agriculture, it causes environmental pollution. Herbicides applied to crops may contaminate water reservoirs, causing harm to human health and herbicide-sensitive crops. It is necessary to have sensors to measure the existence of herbicides and plant response to herbicides. Fortunately, DF offers a promising plant-physiology-based method [37,71]. Herbicides typically function by binding to certain sites on the electron transport chain of PSII and thus affecting electron transport and DF emission [36,37,72]. This makes DF powerful indicator of herbicide stress. A lot of effort has been devoted to this topic. In [73], Katsumata *et al.* developed a DF-based method to measure the effects of Simazine (CAT) and 3,5-dichlorophenol (3,5-DCP) on the growth of a green alga. Li and Xing studied the effect of 3-(3,4-dichlorophenyl)-1, 1-dimethylurea (DCMU) on plant photosynthesis and DF [74]. In [75], Guo and Tan developed a kinetic model to describe the processes of herbicide diffusion into plant tissues and binding to the active sites. Based on the model, a biophotonic method to measure the concentrations of herbicides was developed. The developed method was not affected by differences between leaf samples and can measure herbicide at concentrations as low as 0.5 ppm.

### 2.10. Metal Pollution

Modern industry releases many hazardous metal compounds into soil and water. Physicochemical analyses of such pollution have limitations in detecting the bioavailability of metal compounds and their combined effects on living organisms [76]. Metal compounds can affect diverse metabolic processes of plants, especially photosynthesis. Changes in the DF decay curve and other variables indicated that metal pollution can result in PSII photochemical damage and inhibition of the photosynthesis rate [77,78]. DF therefore can provide an alternative method for metal pollution measurement. In [79], Scordino *et al.* conducted research on using DF to sense metal pollution, in which DF of unicellular green algae samples were measured when different concentrations (10^−5^ to 10^−2^ M) of heavy metals (cadmium, chromium, lead, and copper) were applied. Experimental results demonstrated that DF was sensitive to the presence of metal pollutants. The performed analysis allowed determination of phenomenological relationships between DF and the metal concentration. It was thus concluded that DF-based technique could be a suitable general bioassay of metal contamination. Razinger *et al.* studied heavy metal induced stress in potato leaves with DF imaging technique, which also highlighted the power of DF and confirmed DF imaging could be a very responsive and useful technique for detection of heavy metal pollution [80].

### 2.11. Aquatic Ecosystems

Aquatic ecosystems can provide important resources such as food, medicine, biomass and bioenergy. Monitoring and maintaining a healthy and sustainable aquatic ecosystem is very important. Since DF is a sensitive indicator of photosynthesis and plant growth, researchers have tried to use DF to monitor the condition of aquatic ecosystems. In the work of Krause and Gerhardt [81], three parameters of aquatic biomass were extracted from measured DF: chlorophyll concentration, algae population, and primary production of algae. Because different algal species differ in pigment composition and action spectra of photosynthesis, DF can be used to analyze phytoplankton populations. In [82], Gerhardt and Bodemer used monochromatic light for excitation to distinguish quantitatively between the photosynthetic activity of green algae, diatoms, blue-green algae, and cryotophytes. For the DF-based method, there is no need to prepare samples, and it can be used in the laboratory or on-line to monitor the development of algae assemblage. Prokowski tried to use DF as a tool for monitoring chlorophyll a concentration in phytoplankton [83]. Different from other luminescence methods to determine chlorophyll a concentration [84–86], the DF-based method did not demand frequent and troublesome calibration.

### 2.12. Drought Stress

Irrigation is very important to modern agriculture. Optimized irrigation scheduling is required to regulate the timing and quantity of applied water for sustainable development and maximum return. Traditional irrigation scheduling methods mainly rely on soil moisture measurements; however, soil moisture does not reflect the need of plants and excessive irrigation may cause fertilizer runoff and over-seepage, which leads to fertilizer waste and water pollution [87]. Physiologically-based drought stress evaluation is thus wanted. Drought stress limits photons utilized for photosynthetic reactions and DF. In [88], Guo and Tan modeled DF as an output variable of the PSII photo-transduction system and its dependence on photon utilization rate as affected by water status. From the model, an effective way to define and measure plant water status or deficiency (drought stress) was derived according to PSII photon utilization. The method is easy to implement. Analysis and experiments show that the developed method can be used to evaluate plant drought stress effectively.

## 3. Summary and Future Research

Because DF strongly depends on photosynthetic activities, it has the potential to serve as a versatile tool to measure plant stresses and environmental changes. In previous work, DF has been used for the measurements of photosynthesis rate, plant circadian, plant senescence, nutrients, salt stress, chilling stress, heat stress, acid rain, herbicide, metal pollution, aquatic production, and drought stress. DF shows a lot of promise in providing information on many aspects of plant physiology and environment through a single measurement.

For real monitoring in Nature, DF may be affected by multiple factors as a combination. This brings difficulties for quantitative analysis of stresses or plant-environment interactions from DF measurement. Unfortunately, much existing research on DF is only limited to qualitative analysis and empirical correlation from the measured DF signal directly, which is unlikely to provide clean information on specific stress when multiple stresses affect the plants together. These stresses affect the forward and backward reactions rates differently. Kinetic models of DF generation processes can be built based on photochemical reactions and model parameters reflecting reaction rates and initial concentrations can be estimated from measured data. The estimated reaction rates and initial concentration may be used to differentiate the effect of each individual stress. Special attention should be paid to the uniqueness of model parameters and calibration procedures may be employed to map estimated model parameters to physically meaningful variables.
